# Identification and Quantification of a Toxigenic and Non-Toxigenic *Aspergillus flavus* Strain in Contaminated Maize Using Quantitative Real-Time PCR

**DOI:** 10.3390/toxins8010015

**Published:** 2016-01-04

**Authors:** J. Erik Mylroie, Seval Ozkan, Renuka Shivaji, Gary L. Windham, Michael N. Alpe, W. Paul Williams

**Affiliations:** 1 United States Department of Agriculture, Agricultural Research Service, Corn Host Plant Resistance Research Unit, Mississippi State City, MS 39762, USA; gary.windham@ars.usda.gov (G.L.W.); mnalpe@landolakes.com (M.N.A.); paul.williams@ars.usda.gov (W.P.W.); 2 Department of Plant and Soil Sciences, Mississippi State University, Mississippi State City, MS 39762, USA; so35@pss.msstate.edu; 3 University of North Carolina at Greensboro Molecular Core Lab, University of North Carolina at Greensboro, Greensboro, NC 27412, USA; r_shiva2@uncg.edu

**Keywords:** aflatoxin, *Aspergillus flavus*, PCR, quantification, maize, corn

## Abstract

Aflatoxins, which are produced by *Aspergillus flavus*, are toxic to humans, livestock, and pets. The value of maize (*Zea mays*) grain is markedly reduced when contaminated with aflatoxin. Plant resistance and biological control using non-toxin producing strains are considered effective strategies for reducing aflatoxin accumulation in maize grain. Distinguishing between the toxin and non-toxin producing strains is important in determining the effectiveness of bio-control strategies and understanding inter-strain interactions. Using polymorphisms found in the fungal rRNA intergenic spacer region (IGS) between a toxigenic strain of *A. flavus* (NRRL 3357) and the non-toxigenic strain used in the biological control agent Afla-Guard^®^ (NRRL 21882), we developed a set of primers that allows for the identification and quantification of the two strains using quantitative PCR. This primer set has been used to screen maize grain that was inoculated with the two strains individually and co-inoculated with both strains, and it has been shown to be effective in both the identification and quantification of both strains. Screening of co-inoculated ears from multiple resistant and susceptible genotypic crosses revealed no significant differences in fungal biomass accumulation of either strain in the field tests from 2010 and 2011 when compared across the means of all genotypes. Only one genotype/year combination showed significant differences in strain accumulation. Aflatoxin accumulation analysis showed that, as expected, genotypes inoculated with the toxigenic strain accumulated more aflatoxin than when co-inoculated with both strains or inoculated with only the non-toxigenic strain. Furthermore, accumulation of toxigenic fungal mass was significantly correlated with aflatoxin accumulation while non-toxigenic fungal accumulation was not. This primer set will allow researchers to better determine how the two fungal strains compete on the maize ear and investigate the interaction between different maize lines and these *A. flavus* strains.

## 1. Introduction

Aflatoxin is a toxic and carcinogenic secondary metabolite produced by *Aspergillus flavus* during infection of maize and other crops [1,2]. Due to its harmful nature, aflatoxin contamination levels in maize grain are tightly regulated by the FDA, and grain that exceeds set limits results in an economic loss for maize producers [3]. Thus, efforts have been made to reduce aflatoxin accumulation in maize grain through avenues such as detoxification, biological control, and host plant resistance. Breeding programs have been successful in developing resistant maize germplasm such as Mp313E, Mp715, Mp719, and Tex6 [4,5,6,7,8]. Biological control agents, such as Afla-Guard^®^ and AF36^®^, have been shown to reduce aflatoxin contamination in maize [9,10,11,12,13,14,15]. 

A combined effort that includes the use of resistant maize genotypes and a biological control agent shows promise as an effective strategy in combating aflatoxin accumulation. Therefore, it is important to better understand the interaction between maize and both toxigenic and non-toxigenic *Aspergillus flavus* strains. Studies have shown that a positive correlation exists between the total fungal biomass and aflatoxin accumulation [16,17,18]. Furthermore, recent research has shown that when maize ears were co-inoculated with a toxigenic and non-toxigenic strain that there was significantly less aflatoxin accumulation than in ears only inoculated with a toxigenic strain [13]. To better understand the interaction between maize, toxigenic *A. flavus*, and non-toxigenic *A. flavus*, a method to quantify each strain of the fungus from infected ears is necessary. There have been many studies which have developed tools to successfully differentiate between or quantify *Aspergillus* species as well as toxigenic and non-toxigenic *A. flavus* strains [16,19,20,21,22,23,24,25,26]. However, at the time of this research there was no tool available to simultaneously identify and quantify the different strains of *A. flavus* from co-inoculated ears. 

*Aspergillus flavus* strain NRRL 21882 lacks the entire aflatoxin gene pathway [27]. Therefore, the genes in the aflatoxin pathway, which have been used to identify and quantify *A. flavus* in previous studies, would not have been useful in this experiment. The rRNA gene cluster was thus chosen as a target area due to its high copy number and variability and due to its use in previous studies to quantify and identify *A. flavus* and other fungi in the genus *Aspergillus* [16,19,20,21,22,23,24,25,26,28,29,30].

The goal of this study was to find polymorphisms in the fungal rRNA gene region between a toxigenic (NRRL 3357) and a non-toxigenic (NRRL 21882) strain of *A. flavus* to develop a set of primers that allows for the identification and quantification of a toxigenic and non-toxigenic strain of *A. flavus* using qPCR. *A. flavus* NRRL 21882 was chosen for the non-toxigenic strain because it is the strain that is used as the active ingredient in the commercial biological control agent Afla-Guard^®^ (Syngenta Crop Protection; Greensboro, NC, USA) [31]. After development, the effectiveness of these primers was tested in both laboratory and field experiments to validate their ability to identify and individually quantify the two fungal strains under co-inoculated conditions. Aflatoxin accumulation was analyzed on all treatments and genotypes to examine the effects of co-inoculation on aflatoxin accumulation. 

## 2. Results

### 2.1. Sequencing, Primer Design, and Primer Verification

**Figure 1:**
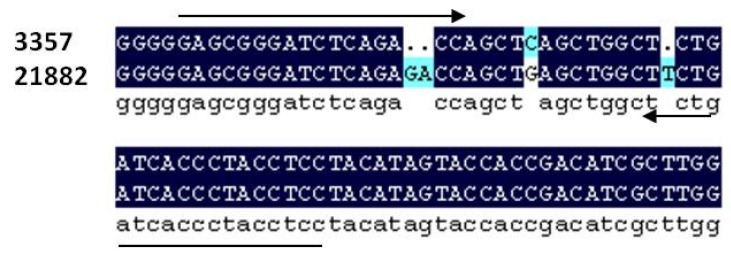
Alignment of section of the IGS region of the rRNA gene complex in *A. flavus* between strain NRRL 3357 and 21882. Alignment shows 2 bp indel used for strain specific primer development as well as other polymorphisms.

**Figure 2:**
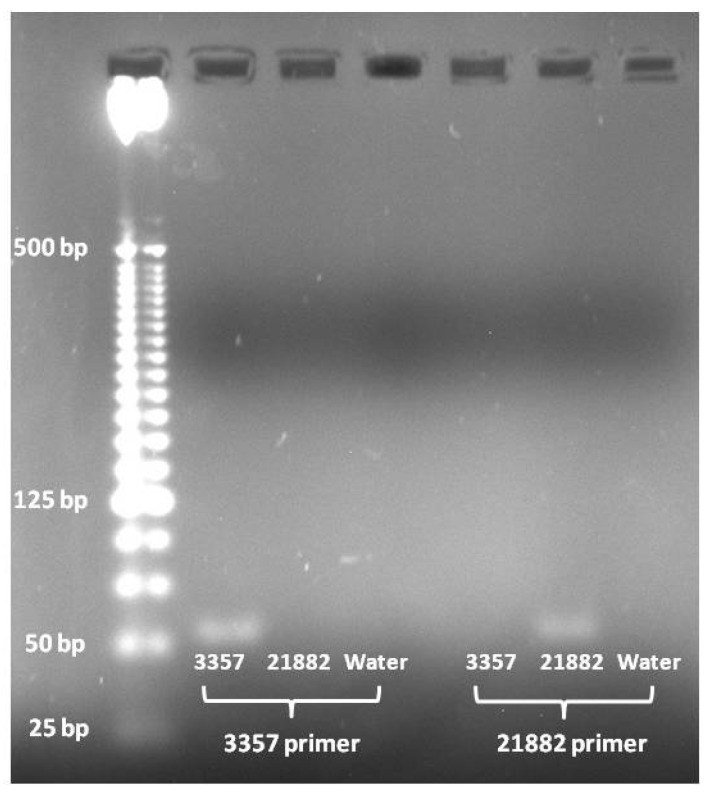
PCR products from *A. flavus* strain specific primers. Lanes contain as follows from left to right: Invitrogen 25 bp ladder, 3357 amplified with 3357 primer pair, 21882 amplified with 3357 primer pair, (−) control amplified with 3357 primer pair, 3357 amplified with 21882 primer pair, 21882 amplified with 21882 primer pair, and (−) control amplified with 21882 primer pair.

Segments of the *A. flavus* rRNA gene complex were sequenced to find polymorphisms that could be used to design primers able to distinguish between NRRL 3557 and NRRL21882. Sequencing using the primers ITS1 and ITS4 revealed no usable polymorphisms in the internal transcribed spacer (ITS) region and therefore the Intergenic spacer (IGS) region was sequenced. Sequencing of the IGS region using the primers LR12R and INVSR1R revealed multiple polymorphisms (Figure 1). A 2-base pair indel between NRRL 3357 and 21882 was used to design primer pairs which amplified an approximately 51 bp fragment (Table 1, Figure 2).

**Table 1:** Primers used for total fungal quantification and strain specific fungal quantification.

Primer Name	Sequence (5′→3′)	Amplicon Size
Af2-F ^a^	ATCATTACCGAGTGTAGGGTTCCT	73 bp
Af2-R ^a^	GCCGAAGCAACTAAGGTACAGTAAA	
3357-F2	GGAGCGGGATCTCAGACC	51 bp
3357-R8	GTAGGAGGTAGGGTGATCAGAGC	
21882-F2 ^b^	GGAGCGGGATCTCAGAGAC	

^a^ Primer pair originally published by Mideros *et al.* 2009 [16]; ^b^ Uses the common reverse primer 3357-F8.

PCR amplification using the 3357 primer pair showed that it only amplified the target fragment in 3357 DNA and not in 21882 or the (−) control (Figure 2). Likewise, the 21882 primer pair only amplified the target fragment in 21882 DNA and not in 3357 or the (−) control (Figure 2).

### 2.2. qPCR Verification

Since the primer pairs successfully demonstrated strain specificity in the PCR tests, the primer pairs were then tested for their efficacy in quantifying the two fungal strains in qPCR conditions and chemistry. An artificial mixed sample test was designed to determine how well the two primer pairs could quantify the two target fungal strains in a mixed sample. The first test was performed using 1 ng/μL of total fungal DNA using 2 test mixtures containing 60% and 40% of each fungal strain and screened using the primers for total fungus (Af2-F & R), 3357 (3357F-2 & 3357R-8), and 21882 (21882F-2 & 3357R-8). Quantification using the Af2 primer pair showed that both mixtures contained a concentration of 1.05 ng/μL of total fungal DNA. In the mixture that contained 60% 3357 and 40% 21882, quantification showed that the sample contained 0.58 ng/μL of 3357 DNA and 0.45 ng/μL of 21882 DNA. The quantification values for the inverse mixture were 0.43 ng/μL of 3357 DNA and 0.65 ng/μL of 21882 DNA (Table 2).

The second test was exactly the same as the first test but was performed using 0.1 ng/μL of total fungal DNA in the 2 test mixtures. Quantification using the Af2 primer pair showed that both mixtures contained a concentration of 0.1 ng/μL of total fungal DNA over the 2 mixtures. In the mixture that contained 60% 3357 and 40% 21882, quantification showed that the sample contained 0.06 ng/μL of 3357 DNA and 0.039 ng/μL of 21882 DNA. The quantification values for the inverse mixture were 0.043 ng/μL of 3357 DNA and 0.063 ng/μL of 21882 DNA (Table 2).

**Table 2:** Quantification of artificially mixed samples of *A. flavus* strains NRRL 3357 and 21882 using qPCR. Strains were mixed in 60:40 ratios at total fungal concentrations of 1.0 ng/μL and 0.1 ng/μL. The Roche LightCylcer^®^ 480 instrument (Roche Diagnostics GmbH, Mannheim, Germany) was used with the SYBR Master kit (Roche Diagnostics GmbH, Mannheim, Germany) and the strain specific primers for 3357 and 21882.

Primer Pair	60% 3357	60% 21882	60% 3357	60% 21882
	0.6/0.4 (ng/μL)	0.6/0.4 (ng/μL)	0.06/0.04 (ng/μL)	0.06/0.04 (ng/μL)
3357	0.575	0.428	0.06	0.0425
21882	0.45	0.65	0.0399	0.0625
Af2 (Total)	1.05	1.05	0.0955	0.1045

### 2.3. Fungal and Aflatoxin Quantification

Analysis of variance showed significant differences between years, among genotypes and among treatments for both aflatoxin and *A. flavus* accumulation (Table 3). All interactions among main effects were significant for aflatoxin but none were significant for *A. flavus* accumulation (Table 3). For genotypes, fungal quantification revealed that in both the 2010 and 2011 field seasons, there were significant differences in total fungus accumulation among the genotypes tested (Table 4). 

**Table 3:** Analysis of variance for aflatoxin and *A. flavus* accumulation in single cross hybrids from the Mississippi State, MS field location in 2010 and 2011.

Source	df ^a^	Mean Squares	
		Aflatoxin ^b^	*A. flavus* Accumulation ^c^
Years	1	24.69 *	5.30 × 10^−4^ *
Reps (Years)	8	1.29	8.40 × 10^−5^
Genotype	3	129.44 *	2.40 × 10^−3^ *
Genotype × Years	3	15.49 *	1.80 × 10^−4^
Reps (Genotype × Years)	24	0.78	2.00 × 10^−4^
Treatment	3	42.35 *	1.20 × 10^−3^ *
Treat × Year	3	17.66 *	1.00 × 10^−4^
Treat × Genotype	9	8.89 *	2.50 × 10^−4^
Treatment × Year × Genotype	9	4.22 *	1.80 × 10^−4^
Error	90	124.60	1.80 × 10^−2^

^a^ df = degrees of freedom. ^b^ Before statistical analysis, means were transformed (ln(*y* + 1), where *y* = aflatoxin concentration). Geometric means expressed in ng/g are the result of a reverse transformation. ^c^ *A. flavus* accumulation measured as ng/μL of DNA. * Significant at α = 0.05.

**Table 4:** Least significant difference (LSD) analysis of fungal accumulation by single cross hybrid across all treatments from Mississippi State, MS field location in 2010 and 2011.

Year	Hybrid	Mean Fungal Accumulation (ng/μL)
**2010**	SC212M × T173	0.0207 ^a^
	GA209 × SC212M	0.0199 ^a^
	Mp313E × Mo18W	0.0047 ^b^
	Mp494 × Mp717	0.0031 ^b^
**2011**	SC212M × T173	0.0144 ^a^
	GA209 × SC212M	0.0115 ^a,b^
	Mp494 × Mp717	0.0039 ^b,c^
	Mp313E × Mo18W	0.0029 ^c^

^a,b,c^ Means followed by the same letter do not significantly differ at α = 0.05.

**Figure 3:**
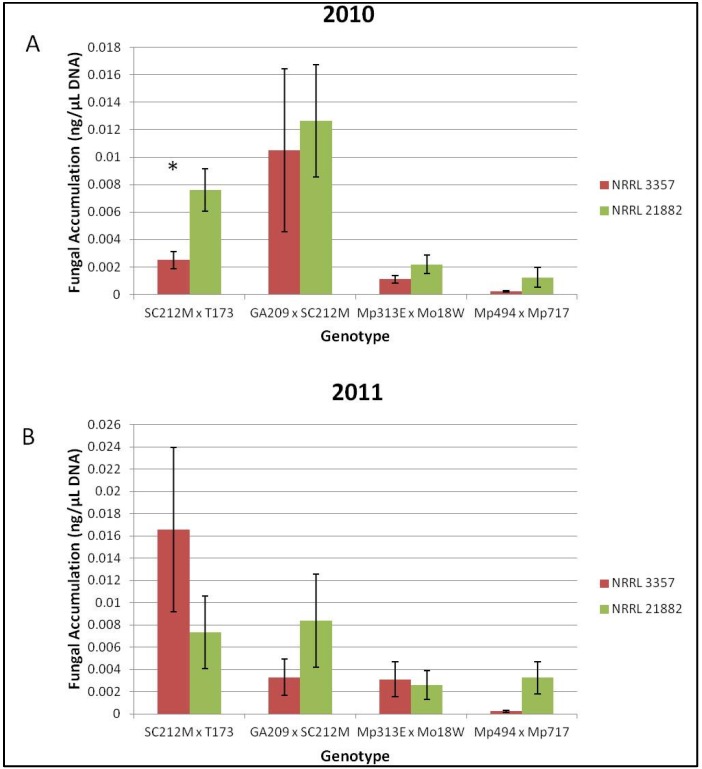
Fungal accumulation (ng/μL) of NRRL 3357 and 21882 in Co-Inoculated ears. (**A**) Fungal strain accumulation for each genotype for the field year 2010; (**B**) Fungal strain accumulation for each genotype for the field year 2011. * Indicates a significant difference (α = 0.05) in *A. flavus* accumulation for strain NRRL 21882 or NRRL 3357 in the Co-Inoculated ears. Error bars represent one standard error.

Overall the resistant hybrids accumulated less total fungus than the susceptible hybrids. However, there were no significant differences among treatments in terms of total fungal accumulation when compared across all hybrids or when compared for each genotype/year combination. When the co-inoculated treatments were quantified using the strain specific primers it was found that neither strain of *A. flavus* was significantly more represented in co-inoculated samples for the means of each genotype in 2010 or 2011. However, when each genotype/year combination was examined, the SC212M × T173 hybrid had significantly more *A. flavus* NRRL 21882 accumulation than NRRL 3357 in the field year 2010 (Figure 3A). No other genotype/year combination had significant differences in strain accumulation (Figure 3A,B). 

**Figure 4:**
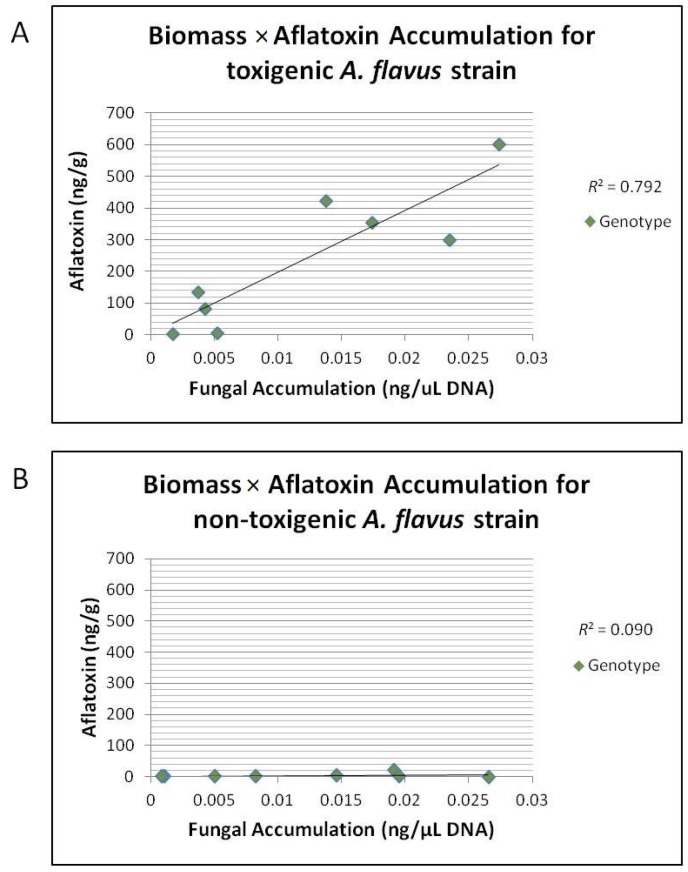
Correlation of mean *A. flavus* strain accumulation (ng/μL) to mean aflatoxin accumulation (ng/g). (**A**) Correlation of mean *A. flavus* strain 3357 fungal accumulation (ng/μL) to mean aflatoxin accumulation (ng/g) in ears for each genotype/year combination inoculated with 3357 or Co-Inoculated for field years 2010 and 2011. There was a strong correlation between 3357 accumulation and aflatoxin accumulation across all genotypes (*R*^2^ = 0.792); (**B**) Correlation of mean *A. flavus* strain 21882 fungal accumulation (ng/μL) to mean aflatoxin accumulation (ng/g) in ears of each genotype/year combination inoculated with 21882. There was a no correlation between 21882 accumulation and aflatoxin accumulation across all genotypes (*R*^2^ = 0.090).

Aflatoxin quantification revealed that for both the 2010 and 2011 field seasons the resistant crosses accumulated significantly less aflatoxin than the susceptible crosses. The geometric means across all treatments for SC212M × T173, GA209 × SC212M, Mp494 × Mp717 and Mp313E × Mo18W were 35, 54, 13, and 2 ng/g respectively in 2010 and 51, 80, 13, and 3 ng/g respectively in the field year 2011 (Table 5). Examination of the differences among the treatments showed that in almost all of the field years, co-inoculation reduced aflatoxin accumulation (Table 6). In regard to treatment across all genotypes, co-inoculation of the two strains reduced aflatoxin accumulation in both years but only significantly in 2010 (Table 7). Correlation for mean *A. flavus* NRRL 3357 fungal accumulation with mean aflatoxin accumulation for each genotype/year combination was highly correlated with an *R*^2^ = 0.79 (Figure 4A); however, there was no correlation between mean NRRL 21882 fungal accumulation and aflatoxin accumulation *R*^2^ = 0.09 (Figure 4B).

**Table 5:** Least significant difference (LSD) analysis of aflatoxin accumulation by single cross hybrid across all treatments from Mississippi State, MS field location in 2010 and 2011.

Single Cross Hybrid	Aflatoxin (ng/g)	
	2010	2011
GA209 × SC212M	54 ^a^	80 ^a^
SC212M × T173	35 ^a^	51 ^a^
Mp313E × Mo18W	13 ^b^	13 ^b^
Mp494 × Mp717	2 ^c^	3 ^c^

Before statistical analysis, means were transformed (ln(*y*+1), where *y* = aflatoxin concentration). ^a,b,c^ Means followed by the same letter do not significantly differ at α = 0.05.

**Table 6:** Least significant difference (LSD) analysis of aflatoxin accumulation by single cross hybrid for each treatment and field year from Mississippi State, MS field location in 2010 and 2011.

Treatment	Single Cross Hybrid							
	SC212M × T173		GA209 × SC212M		Mp313E × Mo18W		Mp494 × Mp717	
	Aflatoxin (ng/g)							
	2010	2011	2010	2011	2010	2011	2010	2011
3357	441 ^a^	233 ^a^	931 ^a^	329 ^a^	115 ^a^	132 ^a^	1 ^a^	3 ^a^
Co-inoculated	102 ^b^	422 ^a^	153 ^b^	92 ^a,b^	16 ^b^	7 ^b^	1 ^a^	4 ^a^
21882	0 ^c^	2 ^b^	0 ^c^	17 ^b^	1 ^c^	0^c^	0 ^a^	0 ^a^

Before statistical analysis, means were transformed (ln(*y*+1), where *y* = aflatoxin concentration). ^a,b,c^ Means followed by the same letter do not significantly differ at α = 0.05.

**Table 7:** Least significant difference (LSD) analysis of aflatoxin accumulation by treatments across all hybrids from Mississippi State, MS field location in 2010 and 2011.

Treatment	Aflatoxin (ng/g)	
	2010	2011
3357	103 ^a^	77 ^a^
Co-inoculated	38 ^b^	33 ^a^
21882	1 ^c^	2 ^b^

Before statistical analysis, means were transformed (ln(*y*+1), where *y* = aflatoxin concentration). ^a,b,c^ Means followed by the same letter do not significantly differ at α = 0.05.

## 3. Discussion

### 3.1. Sequencing, Primer Design, and Primer Verification

Previous studies have used genes in the aflatoxin pathway to identify and quantify toxigenic isolates of *A. flavus* as well as other *Aspergillus* species and other mycotoxin producing fungi [24,26]. However, since the non-toxin producing strain (NRRL 21882) has the entire aflatoxin producing pathway deleted, it was decided to use a different gene for fungal identification and quantification [27]. The rRNA genes were excellent candidates due to their high copy number and variability [21,32]. Based on these criteria, the ITS region was initially chosen for sequencing in order to find polymorphisms useful in primer development. Though used to differentiate among numerous *Aspergillus* species in the past and to quantify total *A. flavus* contamination [16,19,20,21,22,23,24,25,26,28,29,30], sequencing of the ITS region of the *A. flavus* rRNA gene showed no polymorphisms suitable for use in differentiating between NRRL 3357 and NRRL 21882. Therefore, the IGS region was chosen due to its being a region of even higher variability [32]. Multiple indels and SNPs were found between NRRL 3357 and NRRL 21882 and one 2 bp indel was chosen to design primers to differentiate between the two *A. flavus* strains (Figure 1). PCR amplification and visualization showed that the two primer pairs were successful at indentifying the target fungal strain while not producing a product in the non-target strain. 

### 3.2. qPCR Verification

The tests with artificial mixtures of NRRL 3357 and 21882 showed that the primer pair was successful in quantifying each strain in a mixed fungal sample. Quantification results from all of the mixed samples were very close to the 60:40 target ratios when measured (Figure 2). This was a very promising result and showed that the primer pairs could not only successfully quantify a sample composed of a mixture of the two fungal strains but also quantify a mixture with only small differences in concentration with good accuracy. Furthermore, the total fungal amount was near 1.0 and 0.1 ng/μL for the two tests, meaning that, as expected, the Af2 primer set amplified each strain in the mixed sample equally. 

### 3.3. Fungal and Aflatoxin Quantification

Quantification of total fungus showed that in both 2010 and 2011, as expected, the susceptible hybrids (SC212M × T173 & GA209 × SC212M) had more fungal contamination than the resistant hybrids (Mp313E × Mo18W & Mp494 × Mp717). Furthermore, when compared across hybrids there was no significant difference in fungal accumulation among the treatments. Therefore, it can be assumed that under these conditions the genotypes resisted fungal growth at the same rate regardless of which strain was present. When the co-inoculated samples were tested for fungal accumulation using the strain-specific primers, neither strain was found to be significantly more represented than the other strain when compared across all genotypes, and only one genotype/year combination showed a significant difference in strain accumulation (Figure 3). These findings, however, do not confirm or refute the suspected mechanisms of biological control which include competitive exclusion of toxigenic strains by non-toxigenic strains [9,12,33,34,35,36] and intraspecific inhibition [37,38,39,40,41]. More testing in different environments with more genotypes of maize is necessary before any substantive conclusions can be drawn about the interaction between the two fungal strains once infection on the maize ear has occurred.

Aflatoxin quantification analysis showed that the resistant genotypes (Mp313E × Mo18W & Mp494) accumulated significantly less aflatoxin than the susceptible genotypes (SC212M × T173 & GA209 × SC212M) in both the 2010 and 2011 field years. Fungal accumulation and aflatoxin accumulation where highly correlated, as has been found in previous studies [16,17,18]. Furthermore, it was found that in almost all cases (within genotypes and across genotypes) co-inoculated samples accumulated significantly less aflatoxin than those inoculated only with *A. flavus* strain NRRL 3357. The results are consistent with previous research that shows that a biological control agent can be effective in reducing the amount of aflatoxin accumulation in maize ears that are co-infected with a toxigenic fungus [9,13,33,34,35,36,37,38,39,40,41]. 

## 4. Experimental Section

### 4.1. Fungal DNA Extraction and Sequencing

For fungal DNA extraction, *Aspergillus flavus* NRRL 3357 and 21882 strains were grown in 100 mL of potato dextrose broth for 7 days at 28 °C under agitation at 150 rpm. Fungal mycelia were collected via filtration using a Buchner funnel with Whatman™ 110 mm filter paper (GE Healthcare UK; Little Chalfont, Buckinghamshire, UK) then flash frozen in liquid nitrogen. Tissue samples were ground in liquid nitrogen using a mortar and pestle and DNA was extracted using a protocol adapted for volume from Patterson *et al.* [42].

The Internal transcribed spacer (ITS) and Intergenic spacer (IGS) segments of the *A. flavus* rRNA gene were sequenced in the strains NRRL 3357 and NRRL 21882 to identify polymorphisms that could be used to differentiate between the two strains. The ITS region was sequenced using the ITS1 and ITS4 primers and the IGS region was sequenced using LR12R and INVSR1R primers [32]. For the ITS and IGS regions, PCR amplification was carried out using 500 ng of fungal genomic DNA as template in 50 μL reactions with 2 mM MgCl_2_, 0.2 mM of each dNTP, 0.5 μM of the forward and reverse primer and TaKaRa ExTaq DNA polymerase (TAKARA BIO INC.; Otsu, Shiga, Japan). The product was gel purified and was cloned into the TOPO vector using TOPO^®^ TA Cloning^®^ Kit (Life Technologies; Carlsbad, CA, USA). Plasmids with insert were purified from 6 colonies for each line and the insert was sequenced from both directions for all the purified plasmids. Sequencing was performed by the Iowa State DNA Facility (Ames, IA, USA). The sequences from both strains were aligned to look for sequence variations.

### 4.2. Primer Development and Verification

An insertion/deletion was found in the IGS region that could be used to design primers to differentiate between the two strains of *A. flavus* (Figure 1). Two primer pairs were developed to distinguish between *A. flavus* NRRL 3357 and 21882 using Primer3web software v 4.0.0 [43]. The primer pairs shared a common reverse primer, 3357R-8 and two unique forward primers 3357F-2 and 21882F-2 (Table 1). The primer pairs were verified using PCR with a reaction volume of 50 μL that contained 1 μL of template (10 ng/μL), 1 μL 10 mM dNTP Mix, 5 μL 10× PCR Buffer (Sigma-Aldrich^®^; St. Louis, MO, USA), 1 μL of each primer (10 μM), 1 μL of Taq polymerase (Sigma-Aldrich^®^; St. Louis, MO, USA), and 40 μL of H_2_O. The thermocycling steps were: initial denaturation at 94 °C for 2 min followed by 35 cycles of 94 °C for 15 s, 62 °C for 15 s, 72 °C for 30 s, followed by a final elongation step of 72 °C for 10 min. Products were electrophoresed on a 2.5% agarose gel and visualized with ethidium bromide to ensure that the 3357 primer pair only amplified in 3357 and that the 21882 primer pair only amplified in 21882 (Figure 2). Both primer pairs were tested in 3357 and 21882 genomic DNA as well as with a negative control of water.

### 4.3. Standard Curve Development and qPCR Verification

A unique standard curve was developed for use with the two strain specific primers. The standard curve contained concentrations of *A. flavus* as follows: 10, 5, 1, 0.5, 0.1, 0.05, 0.01, 0.005, 0.001, 0.0005, 0.0001, 0.00005 ng/μL of DNA. All serial dilutions of the 5 ng/μL standard point contained 50% of the target *A. flavus* strain and 50% of the other *A. flavus* strain. For example, the 5 ng/μL point in the NRRL 3357 standard curve contained 5 ng/μL of 3357 DNA and 5 ng/μL of 21882 DNA for a total of 10 ng/μL of fungal DNA. All points on the standard curve derived from the 10 ng/μL point contained 100% of the target fungal strain.

Strain specific primers were tested for efficacy in strain differentiation and quantification in qPCR. The Light Cycler 480^®^ instrument from Roche was used with Roche^®^ SYBR green master (Roche Diagnostics GmbH, Mannheim, Germany) for quantification using a reaction volume of 10 μL (5 μL of SYBR green master, 0.5 μL of 10 μM forward and reverse primer, 2 μL of H_2_O, and 2 μL of 10 ng/μL template). The qPCR run was performed as follows: 95 °C for 10 min for initial denaturation, followed by 45 cycles of 95 °C for 10 s, 62 °C for 5 s, and 72 °C for 10 s. The melting curve had once cycle which included 95 °C for 10 s and 65 °C for 1 min followed by a gradual heating to 97 °C with 5 acquisitions per °C. A final cooling step at 40 °C for 10 s concluded the PCR protocol. The two primer pairs were then tested in duplicate using this protocol by quantifying mixed samples of fungal DNA containing both NRRL 3357 and 21882. Test samples containing 60% of one fungal strain and 40% of the other at both 1 ng/μL and 0.1 ng/μL total fungal DNA concentrations were quantified with the 3357 and 21882 primer pairs as well as with primer pair Af2 [16] which quantifies total *A. flavus* concentration (Table 1).

### 4.4. Field Experimental Design, Aflatoxin Analysis and DNA Extraction

Four single cross hybrids were planted in a randomized complete block design at the R. R. Foil Plant Science Research Center located in city, Mississippi State, MS, USA in 2010 and 2011. The genotypes consisted of two susceptible crosses, SC212M × T173 & GA209 × SC212M, and two resistant crosses, Mp313E × Mo18W & Mp494 × Mp717. Ears of each hybrid were inoculated with a toxigenic *A. flavus* fungal strain (NRRL 3357), a non-toxigenic fungal strain (NRRL 21882), water, and co-inoculated with both fungal strains. Inoculation was performed using the side-needle inoculation technique 7 days after mid-silk with a 3.4 mL suspension containing approximately 3 × 10^8^ conidia of the appropriate *A. flavus* strain [44]. Co-inoculation was performed by inoculating the same ear with the conidial suspension of each strain individually at sites in close proximity using the side-needle technique. Primary ears from 10 plants were harvested approximately 60 days after inoculation and were dried for 7 days at 38 °C. 

The ears were then shelled and ground, and aflatoxin accumulation was quantified using the VICAM Aflatest^®^ kit (VICAM, Watertown, MA, USA) using 50 g of ground material. Total DNA was extracted from 100 mg of the same ground material using the GenoGrinder 2000 (OPS Diagnostics LLC; Lebanon, NJ, USA) and the CTAB extraction method [45]. Total DNA was then quantified and diluted to 10 ng/μL for each sample.

### 4.5. Fungal Quantification

Total fungus for all field samples from both 2010 and 2011 was quantified using the Af2 primer pair. All quantification used an in run standard with concentrations of *A. flavus* DNA ranging from 10, 1, 0.1, 0.01, 0.001, and 0.0001 ng/μL of DNA. Individual samples were repeated in duplicate. The qPCR conditions for total fungus were as follows: 95 °C for 10 min for initial denaturation, followed by 45 cycles of 95 °C for 10 s, 59 °C for 5 s, and 72 °C for 10 s. The melting curve had one cycle which included 95 °C for 10 s and 65 °C for 1 min followed by a gradual heating to 97 °C with 5 acquisitions per °C. A final cooling step at 40 °C for 10 s concluded the PCR protocol. 

Strain specific quantification was performed using the two strain specific primer pairs to quantify concentrations of 3357 and 21882. As with total fungus, the samples were repeated in duplicate and used an in-run standard curve. The standard curve contained concentrations of *A. flavus* as follows: 10, 5, 1, 0.5, 0.1, 0.05, 0.01, 0.005, 0.001, 0.0005, 0.0001, 0.00005 ng/μL of DNA. For each strain the 100% standards contained the target strain of interest. The qPCR conditions are the same as those described above in the qPCR validation section. 

### 4.6. Statistical Analysis

Aflatoxin concentration values were transformed as ln(*y*+1), with *y* being the aflatoxin concentration, to provide a more normally distributed data set for statistical analysis. Data analysis was performed using the General Linear Model and means for aflatoxin and fungal concentration were compared using Fisher’s Protected Least Significant Difference (LSD) at α = 0.05. SAS ver. 9.4 (SAS Institute, Cary, NC, USA) was used for statistical analysis.

## 5. Conclusions

Biological control agents have been shown to be effective at reducing aflatoxin accumulation in maize and other susceptible crops [9,10,11,12,13,14,15,46]. However, much is still not understood about the relationship between maize, toxigenic fungi and the non-toxigenic fungi used as biological control agents. It could not be determined from our study whether the reduction in aflatoxin accumulation in co-inoculated ears was due to competitive exclusion or intraspecific inhibition between the two *A. flavus* strains. By developing a pair of primers which can be used to successfully, distinctly identify and quantify a toxigenic *A. flavus* strain (NRRL 3357) and a non-toxigenic strain (NRRL 21882) in co-inoculated ears, we have generated a method that can be used to better understand the relationship between accumulation of *A. flavus* and aflatoxin during co-inoculation. It must be noted that these primers were developed only for these two particular *A. flavus* strains and thus their ability to differentiate among other toxigenic and non-toxigenic strains is currently unknown. Further studies will include determining the usefulness of these primers with other toxigenic and non-toxigenic strains of *A. flavus* as well as with other maize genotypes.
